# Immunization of Cats against Fel d 1 Results in Reduced Allergic Symptoms of Owners

**DOI:** 10.3390/v12030288

**Published:** 2020-03-06

**Authors:** Franziska Thoms, Stefanie Haas, Aline Erhart, Claudia S. Nett, Silvia Rüfenacht, Nicole Graf, Arnis Strods, Gauravraj Patil, Thonur Leenadevi, Michael C. Fontaine, Lindsey A. Toon, Gary T. Jennings, Gabriela Senti, Thomas M. Kündig, Martin F. Bachmann

**Affiliations:** 1 Department of Dermatology, Zurich University Hospital, Wagistrasse 12, 8952 Schlieren/Zurich, Switzerland; franziskazabel@hotmail.com (F.T.); haasstefanie206@hotmail.com (S.H.); Gary.jennings@usz.ch (G.T.J.); 2 HypoPet AG, Moussonstrasse 2, 8091 Zurich, Switzerland; 3 Clinical Trials Center Zurich, University Hospital Zurich, Moussonstrasse 2, 8044 Zurich, Switzerland; alineerhart@hotmail.com; 4 vetderm.ch, Ennetseeklink für Kleintiere, Rothusstrasse 2, 6331 Hünenberg, Switzerland; cnett@vetderm.ch; 5 dermaVet, Tierklinik Aarau West AG, Muhenstrasse 56, 5036 Oberentfelden, Switzerland; s.ruefenacht@dermavet.ch; 6 Graf Biostatistics, Amelenweg 5, 8400 Winterthur, Switzerland; graf@biostatistics.ch; 7 Benchmark Animal Health, Benchmark Holdings Plc, 8 Smithy Wood Dr, Sheffield S35 1QN, UK; arnis.strods@bmkvaccines.com (A.S.); gauravraj.patil@bmkvaccines.com (G.P.); thonur.leenadevi@bmkvaccines.com (T.L.); michael.fontaine@bmkanimalhealth.com (M.C.F.); lindsey.toon@bmkanimalhealth.com (L.A.T.); 8 Director Research and Education, University Hospital Zurich, Rämistrasse 100, 8091 Zurich, Switzerland; Gabriela.senti@usz.ch; 9 Department of Dermatology, University Hospital Zurich, Gloriastrasse 31, 8091 Zurich, Switzerland; Thomas.kuendig@usz.ch; 10 Department of Immunology, Inselspital, University of Bern, Salihaus 2, 3007 Bern, Switzerland; 11 Jenner Institute, University of Oxford, Old Road Campus, Roosevelt Drive, Oxford OX3 7BN, UK

**Keywords:** cat allergy, vaccination, Fel d 1, HypoCat™, virus-like particle

## Abstract

An innovative approach was tested to treat cat allergy in humans by vaccinating cats with Fel-CuMV (HypoCat^TM^), a vaccine against the major cat allergen Fel d 1 based on virus-like particles derived from cucumber mosaic virus (CuMV-VLPs). Upon vaccination, cats develop neutralizing antibodies against the allergen Fel d 1, which reduces the level of reactive allergen, thus lowering the symptoms or even preventing allergic reactions in humans. The combined methodological field study included ten cat-allergic participants who lived together with their cats (*n* = 13), that were immunized with Fel-CuMV. The aim was to determine methods for measuring a change in allergic symptoms. A home-based provocation test (petting time and organ specific symptom score (OSSS)) and a general weekly (or monthly) symptom score (G(W)SS) were used to assess changes in allergic symptoms. The petting time until a pre-defined level of allergic symptoms was reached increased already early after vaccination of the cats and was apparent over the course of the study. In addition, the OSSS after provocation and G(W)SS recorded a persistent reduction in symptoms over the study period and could serve for long-term assessment. Hence, the immunization of cats with HypoCat^TM^ (Fel-CuMV) may have a positive impact on the cat allergy of the owner, and changes could be assessed by the provocation test as well as G(W)SS.

## 1. Introduction

Cats are among the most popular and common pets worldwide and are a significant source of indoor allergens [1]. Hence, allergies to cats are widespread, with a prevalence of 10%–30% in the Western population [2]. A total number of 10 *Feline domesticus* (Fel d) allergens, that are recognized by human IgEs, have been identified [3,4,5,6,7,8,9]. Fel d 1, an uteroglobin-like protein, is considered to be the major cat allergen. In fact, 94% of patients allergic to cats have Fel d 1-specific IgE [10]. Fel d 1 belongs to the family of secretoglobins with homologies to uteroglobin. Its function is unknown but it has been postulated to play a potential role in skin protection and pelt conditioning or have an involvement in the transport of steroids, hormones and pheromones [11,12]. Fel d 1 is produced in sebaceous, salivary, lacrimal, and anal glands and is present in the saliva, tears, skin and fur [13,14,15,16]. It is shed from the cat to the environment through airborne dander and if inhaled by humans may result in sensitization and induction of cat allergy [17]. 

The immune response against innocuous cat allergens is characterized as type I and IV hypersensitivity, involving Th2 cells shaping the environment for production of IgE antibodies by B and plasma cells and recruitment of additional inflammatory cells [18,19,20]. Affected patients suffer from mild symptoms, e.g., sneezing, itchiness of skin and eyes, to severe symptoms ranging from conjunctivitis, rhinitis to asthma, which, upon direct exposure to cats, can lead to life-threatening conditions. There are several recommendations for dealing with cat allergy [21]. Allergic people are advised to avoid allergen exposure by removal of all potential allergen-containing or contaminated objects in the households, e.g., pillows, blankets, carpets, rugs. Environmental cleaning and the use of air humidifiers and HEPA filters can also contribute to the relief of symptoms. Another approach is to remove the cat. However, the bond between owners and their cats is often so strong that they are more likely to accept the risk to their health, which they may not even be fully aware of, than give up their pet [22]. 

Cat allergic subjects usually treat their allergic symptoms with antihistamines and corticosteroids. Another possibility is allergen specific immunotherapy (AIT), which is the only disease-modifying option, but carries the risk of inducing serious side effects and may take years. In fact, AIT can require 30–80 injections over a duration of three to five years with a low chance of success. New approaches explore different routes of administration (e.g., epicutaneous, sublingual, intralymphatic), different formulations of allergens with adjuvants (e.g., MPL, MCT), the introduction of mutations into the protein sequence which delete T cell or IgE epitopes, and finally the use of short peptides instead of full-length allergens [23,24,25,26,27,28,29,30,31]. The challenge for the development of new therapies is exemplified by the recent failure of a phase III clinical study testing a peptide-based vaccine to treat cat allergy [32]. Orengo et al. are developing a monoclonal IgG antibody therapy targeting Fel d 1 in humans that aims to increase the allergen-specific IgG/IgE ratio and relieve symptoms and showed good clinical impact [33]. 

An alternative approach to the problem of cat allergy, and one that does not involve separation of the cat from its owner, is to lower Fel d 1 on the animal itself. One recently described method is the addition of anti-Fel d 1 IgY harvested from chicken eggs to cat food. A reduction in active Fel d 1 in saliva and fur has been reported but whether this will result in clinically significant reductions in allergy still needs to be addressed [34,35]. 

Another approach to lowering allergenic Fel d 1 levels on the cat is active immunization with the aim of inducing anti-Fel d 1 antibodies in the animal itself. Towards this end, a feline vaccine targeting Fel d 1 in cats to treat cat allergy in humans is being developed [36]. The vaccine is based on a recombinantly expressed Fel d 1 protein covalently linked to a virus-like particle (VLP) derived from the Cucumber mosaic virus (CuMV) [37]. The VLP consists of the CuMV coat protein, without any viral genetic information, which serves as a carrier and induces, due to its particulate and repetitive structure, strong and sustained antibody responses, even against self molecules like the Fel d 1 protein in cats [38,39]. To date, vaccination with Fel-CuMV (HypoCat^TM^) has been tested in 70 cats and was well tolerated without short- or long-term (two years) side effects. Furthermore, vaccination induced strong neutralizing anti-Fel d 1 IgG responses lowering levels of reactive allergen in tear extracts of study cats tested with human basophils from cat allergic subjects [36].

In the current manuscript, we report the results of a first field trial with ten cat allergic participants living together with their cats, and the cats were vaccinated with Fel-CuMV. The aim of this exploratory methodology study was 1) to determine a suitable method for measuring a change in the allergic symptoms of the owner and 2) quantify changes in the interactions between the cat and the owner. These methods may then be used in a larger trials in the future. Three parameters were monitored over a duration of almost two years. A home-based provocation test determined the petting time, defined as the time during which the owner was able to interact with the cat until a certain level of symptoms using a visual analogue scale (VAS score of 5) was reached and their organ-specific symptoms score (OSSS) after petting their cats. In addition, a general weekly or monthly symptom score (GWSS and GSS, respectively) assessed overall changes in allergic symptoms in human subjects without interaction with the cat. The provocation test was assessed as a parameter of the acute allergic reaction, comparable to a hospital-based provocation test (e.g., nasal or conjunctival test), whereas the G(W)SS served as readout of chronic symptoms. As observed in previous studies [36], vaccination with Fel-CuMV was well-tolerated by cats and induced strong serum antibody responses. Changes in symptoms of cat owners and interaction times with their cats were observed after vaccination of the pets. Thus, both tests, the provocation test and the G(W)SS, are suitable to detect changes in the symptoms of cat allergic patients. In particular, the petting time may serve as an early efficacy read-out following vaccination of the cats, whereas the OSSS and G(W)SS seem to be more appropriate for long-term monitoring.

## 2. Materials and Methods

### 2.1. Study Population

The study participants were recruited from March till July 2017 and the study commenced in April 2017. Upon completion in February 2018, an extension study with seven of the study participants was conducted from May 2018 until April 2019. Males or females aged 18-65 with a history of cat allergy that were cohabitating with cat(s) were eligible for study inclusion. An understanding of the nature, meaning and scope of the study and signing of an informed consent were also required. Participants were also required to test positive for skin prick tests performed with histamine dihdrochloride and cat allergen extract. In order to confirm the cat allergy to their own cat, participants were also tested positive by a screening scratch test to fur of their own cat. 

Participants were not enrolled if they suffered from immunosuppression or anemias, leukemia or other hematological diseases. Additional exclusion criteria were: pregnancy, breast feeding or intent to become pregnant during the course of the study; a positive skin prick test with the negative control; a known history of anaphylactic reactions to pet allergens; the use of Beta-blockers, neuroleptic drugs and tricyclic anti-depressants. 

Medications like ACE-inhibitors and Beta2-agonists as well as anti-histamines and corticosteroids could influence the study results and were therefore prohibited within 3 days prior to the application of allergen extracts or the provocation test. 

*Main study*: Ten cat allergic participants ranging from 21 to 51 years old, including eight women and two men, were screened and enrolled in the study at the University Hospital Zurich, Switzerland (Table 1). A total of 13 cats (two participants had two or three cats, respectively) were enrolled in the animal part of the study. 

*Extension study*: Seven participants, including six women and one man, aged between 22 and 52 years old continued in the extension study. A total of nine cats (one participant had three cats) were enrolled in the animal part of the study.

### 2.2. Study Design

The study was a single-center, open-label, non-placebo controlled, combined, methodological field trial to determine (a) method(s) for measuring cat allergy symptoms in participants that have immunized their cats with Fel-CuMV. 

**Human part:***Main study*—Screening visits took place at the Clinical Trials Center, University Hospital Zurich (USZ), CH in collaboration with the Department of Dermatology (USZ). After the informed consent was given, the participants underwent two tests to confirm their cat allergy. A skin-prick test was performed using a standard cat allergen and a scratch-test was performed with fur from the own cat. Home-based provocation tests were performed in study weeks 1–3 before vaccination of the cat(s), and in weeks 4, 8, 12, 16, 20 and 24 after vaccination of the cat(s). The provocation test assessed two parameters, the petting time and organ-specific symptoms score (OSSS). The test was only valid if participants had not taken medication containing anti-histamines less than 3 days before the test, otherwise they had to reschedule the test accordingly. If the rescheduling did not happen, the test for that timepoint was invalid and not considered in the statistical analysis. Once a week, the participants filled in a questionnaire retrospectively recording their organ-specific symptoms of the previous week (i.e., general weekly symptom score GWSS). A close-out phone call at the end of the study in week 29 was done to follow up on the well-being and health of the study participant. 

*Extension study* - Seven of ten participants of the main study signed the informed consent form and were enrolled in the extension study. They performed a provocation test before the booster injection of their cat(s) in study week 1 (or week 54 in the combined schedule of main and extension study including the intervening time) followed by four additional tests in study weeks 5, 9, 25 and 45 (weeks 58, 62, 78 and 98, respectively). Again, a provocation test was only valid if participants had not taken medication containing anti-histamines less than 3 days before the test. In the event of a breach, the procedure was followed as described in the main study. In addition, participants recorded their general organ-specific symptoms without provocation monthly (i.e., general specific symptoms GSS). The study finished with a close-out phone call by the clinical study team in study week 45 (week 98, respectively).

**Animal part:** All interventions for the cats were performed by study veterinarians at the “Kleintierpraxis Schwäntenmos” in Zumikon, Zurich, “Ennetseeklinik für Kleintiere AG” in Hünenberg, Zug and “Tierklinik Aarau West AG” in Aarau, Aargau. 

*Main study*—Thirteen cats were enrolled and received three subcutaneous injections of 100 µg Fel-CuMV formulated in 1 mL in study weeks 4, 7, and 10. Sera were collected in study weeks 4, 10, and 27 and analyzed for specific antibody responses. 

*Extension study*—Nine cats received a subcutaneous booster injection of 100 µg Fel-CuMV formulated in 1 mL in week 3 (or week 56 in the combined study schedule). Sera were collected before the boost (week 56), 6 weeks after the boost (week 62) and at the end of the extension cat study (week 78). 

### 2.3. Ethics Approvals

The protocol, participant information and consent form, as well as other study-specific documents, were submitted to the Zurich-based properly constituted cantonal ethic committee (KEK) in agreement with local legal requirements for formal study approval. The decision of the KEK concerning the conduct of the study had been made in writing to the Sponsor-Investigator before commencement of this study. The KEK decided the trial could be conducted without its approval, as the law for human research was judged to be not applicable. From an ethical perspective, the extension study was considered the same and was conducted without particular approval of the KEK. However, both studies were conducted in accordance with ICH/GCP Guidelines and registered with CLINICALTRIALS.gov as NCT03089788.

The Veterinary Offices of the participating cantons Zurich, Zug and Aargau approved the animal study ZH245/16 (19th March 2016). All cat owners signed informed consent for their cats. All interventions and examinations performed by the study veterinarians were in accordance to the Swiss Animal Welfare Ordinance and Animal Welfare Act on Animal Experimentation (2005, TSchG; 2008, TschV). The animal study was designed as an open-label, non-placebo controlled, multi-center, tolerability and immunogenicity study. 

### 2.4. Study Objective

The objective of the study was to determine if the methods tested herein (provocation test and G(W)SS) were suitable to measure changes in allergy symptoms and interaction of the participants with their cats following immunization with Fel-CuMV (HypoCat^TM^). The change in symptoms was assessed at baseline versus week 24 at the end of the main study and over the course of the entire study (main and extension study). The selected method(s) could be used in future clinical trials.

### 2.5. Provocation Test

In an earlier study “ZU_Hyposcore-001” (NCT02399579. https://clinicaltrials.gov/ct2/show/results/NCT02399579) the validity of the provocation test (i.e., HypoScore), a new self-assessed, home-based score specific for cat allergy, was evaluated in cat-allergic participants without vaccination of their cats. In the current study, the provocation test was used to observe changes in allergic symptoms upon vaccination of cats with Fel-CuMV. The test assessed two parameters: the petting time and organ-specific symptoms score (OSSS). The test was performed by petting the cat in order to measure the time until the participant reached a defined symptom strength level (self-assessed, 5 on a VAS ranging from 0–10). If the participant did not reach a symptom level of 5, the petting was stopped at 45 min. When symptom severity of 5 was not reached but provocation time was <45 min (e.g., did not tolerate further petting), for data analysis the time of the last provocation test was carried forward (LOCF). The petting times were measured at baseline on three occasions (week 1–3) before immunization of the cat and over the course of main and extension study. After the provocation test, the participants filled in an OSSS questionnaire on a 4-point scale (0 = no symptoms up to 3 = severe symptoms) regarding their symptoms of eyes, nose, bronchia, lung and palate. Values of the OSSS could be between 0 and 30. The baseline of the HypoScore was measured weekly on three occasions before vaccination of the cats and was compared to the end (week 24) and over the course of the study (weeks 8, 12, 16, 20 and 24 in the main study and weeks 54, 58, 62, 78 and 98 in the extension study) after vaccination of the cats in weeks 4, 7, 10 and booster injection in week 56.

### 2.6. General Weekly or Monthly Specific Symptoms (GWSS or GSS) 

Changes in the general organ specific symptoms on a 4-point scale (0 = no symptoms up to 3 = severe symptoms) of eyes, nose, bronchia, lung, and palate were assessed weekly in the main study (GWSS) or monthly in the extension study (GSS) before and after vaccination of cats with Fel-CuMV. The test was performed without provocation (i.e., direct interaction with their cat). Values of the G(W)SS could be between 0 and 30. The baseline of the general symptoms was determined on three occasions (week 1–3) before vaccination and on 22 occasions during the main study (week 4–25) and 12 occasions during the extension study (week 54-98) after vaccination of the cats in study weeks 4, 7, 10 and booster injection in week 56.

### 2.7. Cat Population

The cats which participated in the study were privately owned and included 13 cats of six breeds including; British Shorthair, European Shorthair, Russian Blue, Ragdoll, Abyssinian, and Egyptian Mau of both sexes (7 ♀; 6 ♂). The age ranged from 4 to 13 years and body weights of 2.5 to 7.4 kg. Thirteen animals were enrolled in the main and nine cats in the extension study. Where several cats lived in the same household, all cats were immunized, but only one cat was designated as the study cat. The cat owner performed the provocation tests and answered all questionnaires regarding the single study cat. 

### 2.8. Vaccination of Cats

The vaccine Fel-CuMV and the vaccine production was described previously [36]. A vaccine dose contained 100 µg Fel-CuMV (HypoCat^TM^) formulated in 1 mL aqueous buffer solution. Subcutaneous injections were applied in study weeks 4, 7 and 10 (main study) and 56 (extension study). The vaccine used in this study was produced by Benchmark Vaccines Limited, UK using a GMP-like production process. Prior to each vaccination, the cats were thoroughly examined by one of the participating veterinarians for their general health status, including a routine physical exam with measurement of the body weight and temperature, checking the site of injection, pulse and breathing rate, abdomen palpation and general appearance. During the course of the study, the owners regularly checked the health of their animal. Twenty-four hours after every injection the owners were called by the study personnel to enquire after the well-being of the cat.

### 2.9. Antibody Responses in Cats

Blood for serum antibody measurements was collected from the vena jugularis or cephalica using serum tubes (Sarstedt, Germany). Sampling was done in the study weeks 4, 7, 10, 27, 56, 62, and 78. After clotting (30 min) and centrifugation (1000 ×*g*, 5 min) of blood samples, sera were transferred to labeled polypropylene tubes (Eppendorf tubes, 1.5 mL, Germany) and stored at ≤ −15 °C until thawed for antibody analyses. 

A validated ELISA (developed by HypoPet AG) was performed to determine anti-Fel d 1 IgG in cat sera previously described [36]. ELISA titers are given as the reciprocals of the dilutions needed to achieve 50% of the optical density of the maximal signal measured at saturation (OD50). The geometric mean titers were calculated from the individual titers of the cats from each group.

### 2.10. Data and Statistical Analyses

The analysis for the human part included data from 9 out of 10 participants and was performed according to the "per protocol" (PP) analysis. Analyses were conducted in this way because one participant had urticaria, accompanied by pruritus and wheal formation, on two occasions during the study. The participant was unable to distinguish urticaria symptoms from the symptoms of cat allergy and was thus excluded from analysis. The analyses of cat data included all 13 cats, whereas the correlation of antibody responses in cats with measurements of human allergic symptoms included data from 9 out of 10 participants with their respective study cats (*n* = 9).

Statistical analyses were performed with Excel, Graph Pad Prism and R. Data were summarized by descriptive statistics: mean, standard deviation, median, first and third quartile, minimum, and maximum. This study was classified as a pilot study because there was no pre-existing information available concerning the expected change in symptoms of allergic cat owners before and after immunization of their cats (the effect size). The changes in the symptoms of the main study were evaluated using an exact Wilcoxon matched-paired signed rank test. Hypotheses were tested at a two-sided significance level of α = 0.05. For comparison between baseline and course of study, the mean of all values was computed.

## 3. Results

### 3.1. Study Design and Population

**Figure 1:**
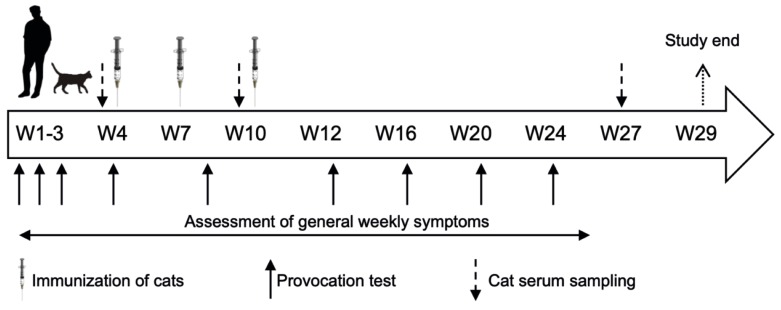
Study design of the combined human and animal trial. Prior to immunization of their cats, participants performed a baseline assessment of their allergic symptoms using the HypoScore (OSSS and petting time) and GWSS. In the course of the study, they performed the provocation test every four weeks and recorded their general symptoms weekly without provocation using the GWSS. Cats received three injections of 100 µg Fel-CuMV (HypoCat^TM^) at intervals of three weeks (in study weeks 4, 7 and 10) subcutaneously. Cat sera were collected at baseline, week 10, and week 27.

This trial was an open-label, non-placebo controlled, single-center, exploratory study involving both cat-allergic humans and their cats (Figure 1). The aim of this methodology field trial was to assess different tests to measure allergic symptoms and to monitor possible changes in allergic symptoms in the study participants after their cats were immunized with the cat vaccine Fel-CuMV.

The study included 10 human participants (Table 1), ranging from 21 to 51 years old, and 13 cats (one participant had two cats and another three cats), ranging from 4 to 13 years old, of both sexes and six different breeds. All ten participants who were screened were enrolled in the study. There were no drop-outs and 10 of 10 participants completed the study. One participant suffered from recurrent chronic urticaria of unknown origin, but exhibited no symptoms at the beginning of the study. However, the urticaria occurred in study week 2 and again in week 16. On each occasion the urticaria lasted for 4–5 weeks until recovery. The participant was not able to distinguish symptoms of urticaria from those of cat allergy and was excluded from the analysis. Data are thus presented as “per protocol” (PP) analysis. 

### 3.2. Adverse Events

Since there was no treatment of the participants, the risk of inducing study-related adverse events was considered low. Nevertheless, some adverse events were recorded and included a cat bite during a veterinary visit, chronic urticaria, erythema, common cold, and pruritus (Table 2). The participant bitten by their cat during an immunization procedure at the veterinary practice was referred to their doctor and received prophylactic antibiotic treatment. They fully recovered from the wound after 1 week. Another participant #10 (excluded from analysis) suffered from an urticaria and presented with strong pruritus and wheal formation. The symptoms were treated with topical urea, macrogol-6-laurylether and fexofenadine-hydrochloride.

There were no severe adverse events observed in the cat population. Several minor clinical signs were noted upon immunization with Fel-CuMV and were judged to be normal vaccination reactions in cats. They disappeared within 72 h of administration. All reactions during and after injection of Fel-CuMV were less frequent on the second and third dosing occasions. There were no treatment related changes in body weight or temperature observed over the study period. Cat owners reported no change in the behavior or appearance (e.g., fur condition) of the cats. 

### 3.3. Assessment of the OSSS after Provocation

**Figure 2:**
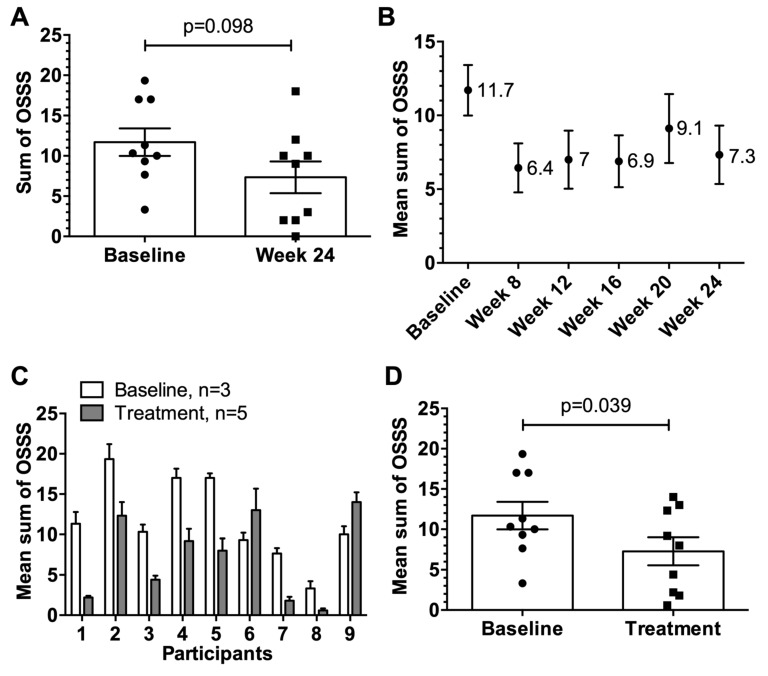
Organ specific symptom score (OSSS) after provocation. (**A**) Individual OSSS with SEM of participants (*n* = 9) at baseline versus week 24 at the end of the study. (**B**) Mean OSSS with SEM of participants (*n* = 9) at baseline and over the course of the study. (**C**) Individual mean OSSS with SEM comparing baseline (weeks 1–3, *n* = 3) vs. treatment period (weeks 8, 12, 16, 20 and 24, *n* = 5). (**D**) Individual mean OSSS (*n* = 9) with SEM comparing baseline (*n* = 3, weeks 1–3) vs. treatment period (*n* = 5, weeks 8, 12, 16, 20 and 24). Statistical significances were obtained by an exact Wilcoxon matched-paired signed rank test. Possible OSSS values from 0–30.

Seven of nine participants showed lower OSSS at week 24 compared to baseline (Figure 2A) demonstrating a change in the OSSS after provocation. The change from mean 11.7 at baseline to mean 7.3 at week 24 was not significant (*p* = 0.098). The change in the OSSS over the course of the study was also assessed (Figure 2B–D). There was a significant reduction in the mean OSSS from week 8 to 24 (mean OSSS ranging from 6.4–9.1) compared to baseline (mean OSSS 11.7) (Figure 2B). The reduction in the OSSS in seven of nine participants over the study period compared to baseline was statistically significant (*p* = 0.03) (Figure 2C,D), demonstrating a sustained reduction in organ-specific symptoms after vaccination of the cat observed from week 8 to week 24.

### 3.4. Petting Time 

**Figure 3:**
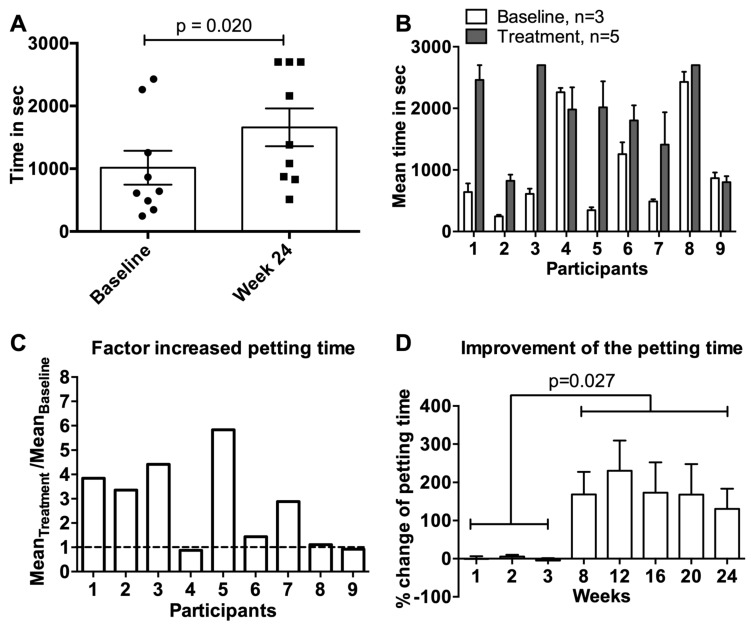
Time of petting as part of the provocation test. (**A**) Mean petting time with SEM in seconds of nine participants comparing baseline (*n* = 3) versus week 24 at the end of the study. (**B**) Individual mean petting time with SEM in seconds at baseline (*n* = 3, weeks 1–3) compared to treatment period (*n* = 5, weeks 8, 12, 16, 20 and 24). (**C**) Factor of changed petting time for each participant shown as ratio of the mean timepoints during treatment (*n* = 5, weeks 8, 12, 16, 20 and 24) to mean baseline time (*n* = 3, weeks 1–3). (**D**) Change in % of the mean petting time with SEM of nine participants during the treatment period in weeks 8, 12, 16, 20 and 24 compared to mean baseline in weeks 1–3. Statistical significances were obtained by an exact Wilcoxon matched-paired signed rank test.

There were eight out of nine possible successes, meaning that the time of interaction with their cats to reach a defined level of allergic symptoms for eight participants was greater at week 24 than the mean baseline of weeks 1–3 (Figure 3A). The change from mean 16.9 min (1016 s) at baseline to mean 27.7 min (1659 s) was statistically significant (*p* = 0.02). Of note, three participants had reached the maximum interaction time of 45 min by the end of the study. 

The improved petting time was apparent over the entire course of the study after vaccination of the cats in weeks 4, 7 and 10 (Figure 3B–D). On a per participant basis, the petting time was increased in seven of nine participants up to 6-fold (Figure 3B,C), which corresponded to an average improvement of 100%–230% across weeks 8, 12, 16, 20, and 24 compared to baseline (Figure 3D). 

### 3.5. General Weekly Symptom Score

**Figure 4:**
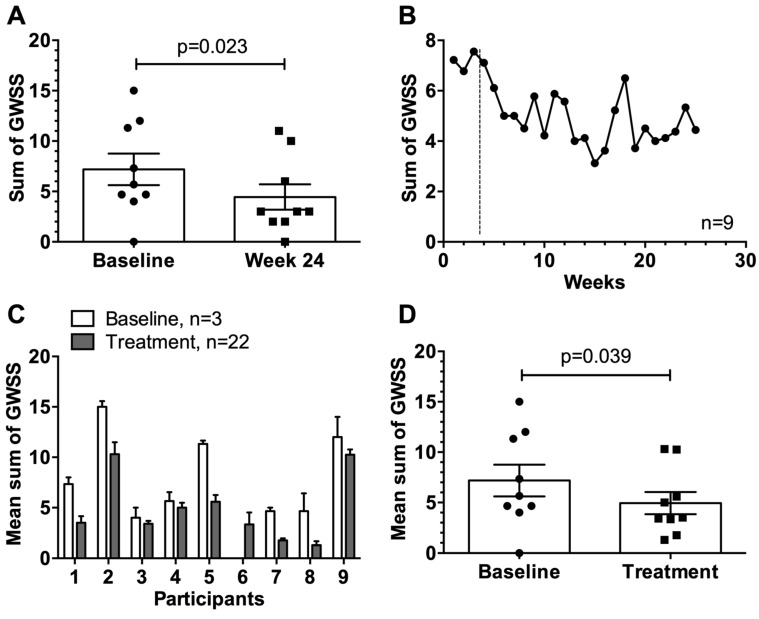
General weekly symptom score GWSS. (**A**) Mean GWSS with SEM of participants (*n* = 9) at baseline (*n* = 3, mean of weeks 1–3) versus week 24 at the end of the study. (**B**) Mean GWSS with SEM of participants (*n* = 9) at baseline (*n* = 3, weeks 1–3) and over the course of the study (*n* = 22, weeks 4–25). (**C**) Individual mean GWSS with SEM at baseline (*n* = 3, weeks 1–3) compared to treatment period (*n* = 22, weeks 4–25). (**D**) Mean GWSS with SEM of nine participants comparing baseline (*n* = 3, weeks 1–3) vs. treatment period (*n* = 22, weeks 4–25). Statistical significances were obtained by an exact Wilcoxon matched-paired signed rank test. Possible GWSS values from 0–30.

There were eight of possible nine successes, meaning that the GWSS of eight participants were lower at week 24 compared to baseline (Figure 4A). The mean GWSS at baseline compared to week 24 was significantly reduced from 7.2 to 4.4 (*p* = 0.023). 

The average GWSS of all participants over the entire study period was also assessed (Figure 4B–D). Towards the end of the study, the mean GWSS was consistently lower than the baseline values (Figure 4B). Comparing the average baseline with the average treatment values of the GWSS, a change in the general allergic symptoms (*p* = 0.039) was observed in eight of nine participants, demonstrating a persistent improvement in symptoms upon immunization of the cats (Figure 4C,D). 

### 3.6. Induction of Anti-Fel d 1 IgG Antibody Responses in Cats upon Vaccination with Fel-CuMV

**Figure 5:**
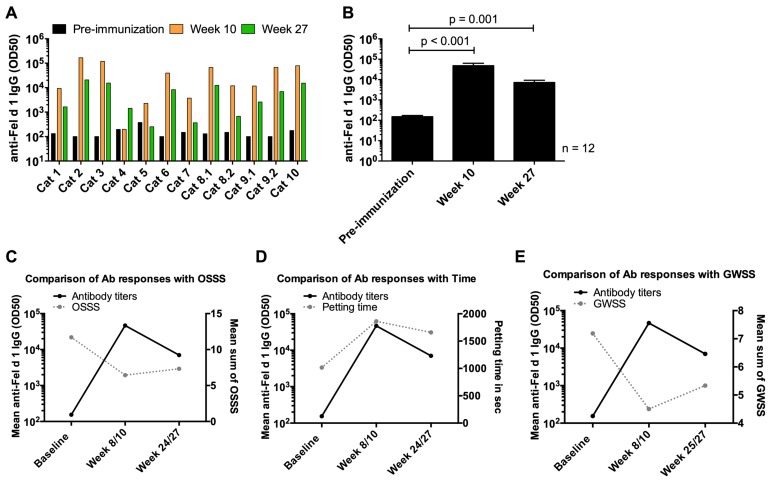
Antibody responses in cat sera. (**A**) Anti-Fel d 1 IgG antibody titers in cats before and after immunization, *n* = 12. (**B**) Mean anti-Fel d 1 IgG antibody titers with SEM, *n* = 12. (**C**) Anti-Fel d 1 IgG antibody titers in cats at baseline (before immunization), week 10 and 27 vs. OSSS at corresponding timepoints baseline (before immunization), week 8 and 24. (**D**) Anti-Fel d 1 IgG antibody titers in cats at baseline (before immunization), week 10 and 27 vs. petting time at corresponding timepoints baseline (before immunization), week 8 and 24. (**E**) Anti-Fel d 1 IgG antibody titers in cats at baseline (before immunization), week 10 and 27 vs. GWSS at corresponding timepoints baseline (before immunization), week 8 and 25. (C-E) Data included from participants (*n* = 9) with corresponding study cats (*n* = 9). Statistical significances were obtained by an exact Wilcoxon matched-paired signed rank test.

Subcutaneous injection of Fel-CuMV vaccine was considered to be well-tolerated and safe, as only mild and transient side reactions were noted. Cat sera were assessed for Fel d 1-specific IgG antibody responses throughout the course of the study (Figure 5). Participant #8 had three cats, identified as cats 8.1, 8.2, and 8.3. There are no serological data available for cat 8.3 because it rejected the sampling procedure. There was a significant increase in anti-Fel d 1 IgG in all vaccinated cats (*p* < 0.001) detectable after two immunizations at week 10. By the end of the study at week 27, the anti-Fel d 1-specific antibody response in sera (*p* = 0.001) was still significantly increased compared to baseline. 

The antibody responses of the study cats were compared with the OSSS and petting time of the provocation test and the GWSS of the participants at the corresponding timepoints (Figure 5C–E). The parameters for assessing the allergic symptoms of the participants correlated well with the antibody responses in cats. Petting time showed a direct correlation with the anti-Fel d 1 IgG titers, whereas the OSSS and the GWSS measurements were inversely correlated with the anti-Fel d 1 IgG titers.

### 3.7. Data of the Extension Study

**Figure 6:**
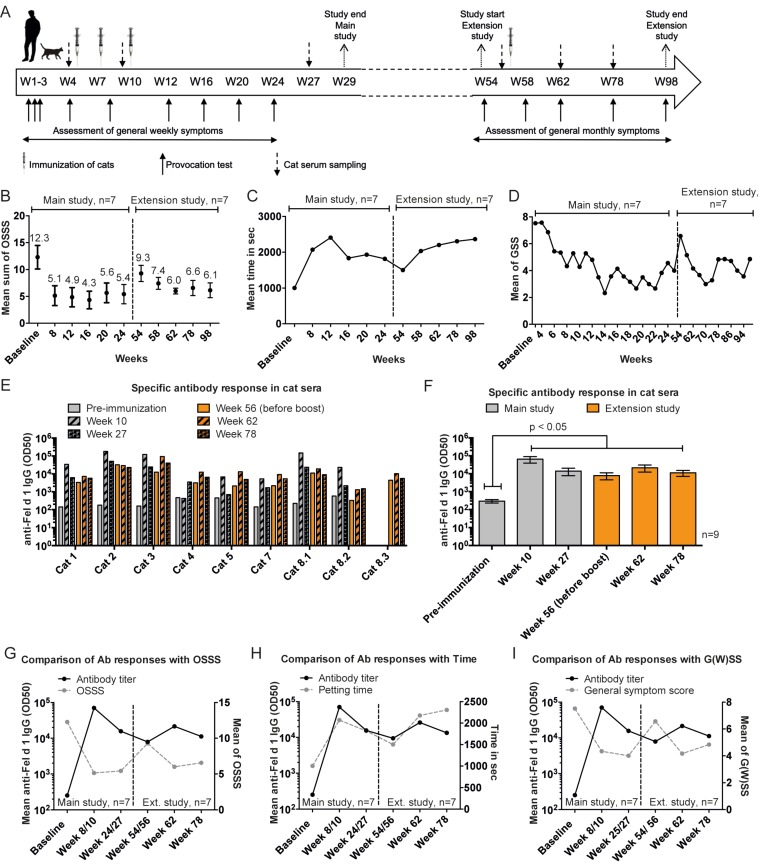
Extension study: Changes in allergic symptoms of participants after booster injection of cats. (**A**) Study design of the main and extension study including seven participants and nine cats. In addition to the main study, the participants performed a provocation test before the boost (week 56) followed by four additional provocation tests in study weeks 58, 62, 78, and 98. Participants also recorded their general organ specific symptoms (GSS) without provocation at monthly intervals during the extension study. The cats received a boost immunization of 100 µg Fel-CuMV subcutaneously in week 56. In addition to the collected serum samples of the main study, cat sera were also collected in study weeks 56, 62, and 78. (**B**) Mean OSSS with SEM over the course of the main and extension study of seven participants. (**C**) Mean petting time over the course of the main and extension study of seven participants. (**D**) Mean general symptoms (G(W)SS) over the course of the main (assessed weekly) and extension (assessed monthly) study of seven participants. (**E**) Measurement of anti-Fel d 1 IgG in cat sera of nine cats participating in the main and extension study. (**F**) Mean anti-Fel d 1 IgG antibody titers with SEM in cat sera. (**G**) Mean anti-Fel d 1 IgG antibody titers in cats vs. mean OSSS over the course of the main (baseline (before immunization), week 8/10 and 24/27) and extension study (week 54/56 (before boost), week 62 and 78). (**H**) Mean anti-Fel d 1 IgG antibody titers in cats vs. mean petting time over the course of the main (baseline (before immunization), week 8/10 and 24/27) and extension study (week 54/56 (before boost), week 62 and 78). (**I**) Mean anti-Fel d 1 IgG antibody titers in cats vs. mean G(W)SS over the course of the main (baseline (before immunization), week 8/10 and 24/27) and extension study (week 54/56 (before boost), week 62 and 78). (**G**-**I**) Data included from participants (*n* = 7) with corresponding study cats (*n* = 7). Statistical significances were obtained by an exact Wilcoxon matched-paired signed rank test.

After completion of the main study, an extension study was conducted with seven of the original participants (Figure 6A). Reasons for the discontinuation of three participants were the exclusion of the urticaria patient, the cat of one participant died due to a study-unrelated icterus, and personal preferences of one participant. 

The analysis presented in Figure 6 includes the data obtained from the seven participants and their respective cats (seven study cats plus two cats; one participant had three cats), who participated in both studies.

As described above, there was a reduction in the OSSS from week 8 to 24 (mean OSSS ranging from 4.3 to 5.6) compared to baseline (mean OSSS 12.3). The mean OSSS increased to 9.3 in the intervening period between both studies (i.e., from week 24 to 54). After the boost immunization in week 56, there was a small decrease in mean OSSS observed in study weeks 58 to 98 ranging from 6.0 to 7.4. Although the reduction in the OSSS over both study periods compared to baseline was not statistically significant, a trend of reduced OSSS was observed over the entire study period of almost 2 years (94 weeks) after vaccination of the cats.

Relative to baseline, the petting time of all seven participants had increased by week 8 after two immunizations administered in weeks 4 and 7 (Figure 6C). In fact, six of seven participants achieved the maximum interaction time of 45 min by week 12. Thereafter, the mean petting time decreased slightly, but still remained higher than the mean baseline petting time. In the intervening period between both studies, the mean interaction time decreased further. However, upon administration of the booster injection in week 54, the interaction time increased again and six of seven participants achieved the maximum time (i.e., 45 min) until study completion in week 98. 

The GSS followed a consistent trend of lower symptoms from week 5 to 25 after the initial cat immunizations in weeks 4, 7 and 10 (Figure 6D). The mean GSS at baseline (weeks 1–3) of 7.5 improved to a mean score of 6.8, which was already detectable in week 5, and further improved to 2.3 in study week 14. By the end of the main study in week 25, the mean GSS of 4 was still lower compared to baseline (i.e., GSS 7.5). Before cats received the boost injection in week 56, the mean GSS increased to 6.5 but was still lower compared to baseline (i.e., GSS 7.5 of weeks 1–3). Upon booster injection, allergic symptoms improved again, reaching the lowest mean GSS of 3 in week 70. Clinical improvement was still evident by the end of the study in week 98 (i.e., GSS 4.8). Notably, the participants had reduced GSS at any timepoint after the first vaccination of their cats in study week 4, as the mean GSS over both study periods was always lower compared to baseline (weeks 1–3).

Cat sera were assayed for Fel d 1-specific IgG antibodies throughout both study periods (Figure 6E,F). The mean Fel d 1-specific IgG titers increased ~300-fold measured after two injections in week 10 and were still on a high level at the end of the main study at week 27 (Figure 6F). Specific antibodies declined between weeks 27 and 56 (before the boost) compared to the peak response detected in week 10. Following administration of a booster injection in week 56, the specific IgG levels increased again in eight of nine cats when measured by week 62, 6 weeks after the boost. Almost 4 months later, by the end of the study in week 78, the anti-Fel d 1 IgG responses were still significantly higher compared to baseline and were slightly above the antibody level measured before boost (week 56). 

The antibody responses were inversely related to the OSSS and G(W)SS over the period of the main and extension studies (Figure 6G,I). In contrast, antibody responses directly correlated to the petting time of the provocation test (Figure 6H), indicating that specific antibodies reduced symptoms and increased petting time. Specifically, after the induction of anti-Fel d 1 antibodies detected in week 10, the OSSS decreased from a mean score of 12.5 at baseline to 5.1 and 5.4, measured in week 8 and 24, respectively. During the intervening phase of the main and extension study, the antibody titers declined and the mean OSSS increased to 9.3, detected in week 54. After the boost injection which caused an increase in specific antibodies, the mean OSSS decreased to a mean score of 6.0 and 6.6 in study weeks 62 and 78, respectively. Similar observations were made for G(W)SS.

The antibody titers also showed a correlation with petting time. Relative to baseline (week 1–3), mean petting time and anti-Fel d 1 IgG, determined in week 8 and 10, increased the mean petting time by a factor of 2, demonstrating that owners were found to be able to interact with their cats twice as long before they developed an allergic symptom strength of 5 according to VAS. The subsequent decrease in specific antibodies was associated with a decrease in petting time observed in weeks 24 and 54, although still above baseline. Upon the increase in specific antibodies after the booster injection, petting time increased again by a factor of 2.3 compared to baseline. 

## 4. Discussion

Allergy is the most common chronic disease in Europe and U.S and often manifests with chronic conditions including rhinitis, conjunctivitis and asthma, which is associated with individual morbidity and high socio-economic costs. In fact, more than 150 million Europeans are affected by chronic allergic diseases (EAACI, 2016). The danger of developing chronic asthma is particularly high, especially in allergies, which are caused by air-borne allergens such as hay fever and cat allergy. One in five patients with allergies lives with the constant fear of getting an asthmatic or anaphylactic shock at any time or even dying as a result of a severe allergic reaction upon allergen encounter. The World Health Organization estimates that approximately 300 million individuals currently suffer from asthma worldwide and expect an increasing incidence to 400 million by 2025 [21,40]. This development is worrisome, threatening health and economies alike, and demands action of the global community for the development of new therapies, medications and diagnostic tools to address this major challenge [7,41]. Cat allergy contributes to a significant proportion of the allergic disease burden. 

There are several new therapeutic developments to treat cat allergy that are currently pursued by various research groups and clinicians. Our novel approach of vaccinating the cat against Fel d 1, offers a cost-effective therapy without the risk of inducing severe side effects in cat-allergic patients, as they often occur during AITs, and without adversely affecting the cat. The vaccine targets Fel d 1, the major cat allergen for humans. By immunizing cats, the reactive allergen level can be lowered, thereby alleviating the symptoms of cat allergic patients. Here, we present clinical data of a first combined human and animal field trial conducted as an open-label, exploratory methodological study. The aim of the study was to assess different methods of measuring the allergic symptoms and determine if those methods could detect changes in allergic symptoms after vaccination of cats with Fel-CuMV (HypoCat^TM^). 

The collection of clinical research data must be reproducible, valid and traceable using standardized procedures [42,43]. However, the selection and combination of various tests and scoring systems for monitoring allergic symptoms and their changes usually have a significant impact on the outcome of clinical trials and the development of innovative medications. Therefore, it is advisable to select as few questionnaires, tests and scoring systems as possible to capture the changes that best reflect the effect of the treatment [44,45]. To this end, we developed a new method to record allergic symptoms by a self-assessed, home-based symptom score, the provocation test. The validity of the test was evaluated in a previously conducted human clinical trial involving non-immunized cats (NCT02399579). The test consists of two elements: an assessment of organ-specific symptom score (OSSS) after petting—a standardized score—and measurement of the time of petting the cat until a defined level of allergy symptom was reached (level of 5 on a VAS). Both parameters, the OSSS and the time, are analyzed separately. In addition to the provocation test, another self-assessed, home-based standard method was investigated in this study: a general symptom score (G(W)SS) assessing the allergic symptoms without provocation. 

The mean OSSS as part of the provocation test at week 24 was reduced in seven of nine participants compared to baseline. Moreover, the change in the OSSS was apparent throughout the course of the main study (weeks 8, 12, 16, 20 and 24 vs. baseline) and showed a variable yet sustained reduction in seven of nine participants. It should be noted that a reduction in the OSSS was not necessarily expected to be large, since the cat owners were required to record their symptoms after petting the cat until they had reached a symptom strength of 5 on the VAS. Hence, upon cessation of the provocation test (i.e., petting), the OSSS would have been similar on each occasion. A reason why a substantial decrease in OSSS in the main study was achieved may have been due to the fact that three participants, at week 24, were able to pet their cat for the maximum time of 45 min. In this circumstance, the VAS score of 5 was not reached, and thus the OSSS was lower. Regarding the extension study, several participants at several occasions could pet their cat to the maximum petting time of 45 min and did not reach the symptom strength of 5 on a VAS. Thus, the allergic symptoms were not as pronounced, resulting in lower OSSS.

The second parameter of the provocation test was the petting time. Relative to baseline, an increased time of petting was observed in eight of the nine participants at week 24 and over the entire period of the main study in seven of the nine. The increase in petting time was even more pronounced in the extension study. Several participants could pet their cat to the maximum time of 45 min on several occasions. The average petting time increased by a factor of two upon immunization of the cats and showed a sustained improvement over the period of the main and extension studies, demonstrating that the participants could interact longer with their cats. 

A change in general allergic symptoms without provocation assessed by the G(W)SS was noted upon vaccination of the cats. Relative to baseline, a reduction in the GWSS at week 24 and over the period of the main study was observed in eight of nine participants. Moreover, the GSS measured throughout the course of the extension study showed a variable yet sustained reduction in seven of seven participants. Of note, the improvement in general allergic symptoms of the participants recorded without direct interaction with their cats at all timepoints after immunization of the cats over the period of the main and extension studies indicates that the participants generally felt better and suffered less from allergic symptoms. 

Subcutaneous injection of three doses of Fel-CuMV vaccine followed by a single boost injection a year later was considered to be well tolerated in several breeds of privately owned adult cats. No serious adverse event occurred during the study. The clinical signs and reactions upon vaccination with Fel-CuMV were mild and reversible. No related effects on body weight, food consumption, behavior and appearance were observed. Moreover, the intended immunological response in cats immunized with Fel-CuMV, i.e., induction of anti-Fel d 1 IgG antibodies, was achieved. Anti-Fel d 1 antibodies were measured in sera at week 10, three weeks after the second immunization. Previous studies in cats (*n* > 60) with Fel-CuMV have shown that about 50% of immunized animals achieved peak titers 2–3 weeks after the second immunization [36]. This would correspond to week 10 of the current study. These findings support the observation of the improved symptoms assessed by the provocation test and G(W)SS of the participants from week 8 and 6, respectively onwards, as Fel d 1-specific antibodies were present and had the potential to neutralize Fel d 1 as previously shown [36]. The improvement of symptoms had lessened by the end of the main study, which may be related to the kinetics of the antibody response, which showed a decline from week 10 to week 27. Upon administration of a booster injection, the symptoms and petting time improved again.

However, the results and conclusions of this open label, exploratory methodology study with a small sample size (*n* = 9 in main and *n* = 7 in extension study) must be taken with caution. There was no pre-existing information available regarding the expected change in symptoms of the participants before and after immunization of their cats. As a consequence, no sample size calculation could be done beforehand. Nevertheless, it is interesting to note that several parameters measured in the study were suggestive that targeting the major cat allergen Fel d 1 by immunization of cats with Fel-CuMV had a positive impact on the allergic sensation of the participant. In particular, the time that participants were able to interact with their cats before a particular level of allergic symptoms was reached increased significantly. 

Improvements of the organ specific symptoms with (OSSS) and without provocation (G(W)SS) over the course of the study were also noted. Notably, it was observed that the improvement in these measures had lessened at the end of the main study but increased again after the booster injection. This observation of the kinetics is valuable for planning and conducting further studies. Therefore, all measured parameters (i.e., provocation test and GSS) are suitable to assess changes in allergic symptoms of the cat owners. 

Finally, from a veterinary perspective, targeting Fel d 1, the major cat allergen in humans, by active vaccination has, to date, been well-tolerated. The general health status of the cats was not compromised by the induction of auto-antibodies against Fel d 1. However, allergic symptoms of the cat allergic owners were alleviated. As a result of the owner being less burdened by their allergy, the quality of life of their cat may be improved. The ability of allergic cat owners to better tolerate and increase the duration of their interactions with their pet can benefit the animal through better training and socialization and awareness of the animals’ overall health. Moreover, the likelihood of abandonment and subsequent euthanasia in animal shelters could be decreased.

## Figures and Tables

**Table 1:** Study population.

Participant#	Age	Sex	Age When Allergy Was Observed the First Time	Affected Organs				Baseline VAS Score (0–10) Without Direct Interaction with Cat	Maximal VAS score (0 –10) After direct interaction with cat	Continued in Follow up Study
				Eyes	Nose	Lungs/Bronchia	Palate			
1	50	female	10	x			x	2.5	8.8	x
2	29	female	8	x	x	x		3.5	9.2	x
3	21	male	21	x	x	x		3.9	7.2	x
4	43	female	28	x		x		1.1	7	x
5	24	female	22	x	x		x	3.7	7.2	x
6	45	male	23	x		x		4.2	9	-
7	40	female	10	x	x			1.3	5.6	x
8	28	female	12	x	x			0.3	7.7	x
9	51	female	41	x	x	x		1.4	8.5	-
10	37	female	23		x	x		1.9	4.9	-
**Mean/Aver.**	**36.8 ±10.8**	**8 ♀; 2 ♂**	**19.0 ± 11.3**	**9/10**	**7/10**	**6/10**	**2/10**	**2.4 ± 1.4**	**7.5 ± 1.4**	**7/10**

**Table 2:** Adverse events.

Adverse Event	Severity	Participant #	Total Number	Percentage of All AEs	Related to Intervention
Erythema and pruritus	mild	9	3	17.6%	Possible
Tooth infection	moderate	9	1	5.9%	No
Common cold	mild	2, 4, 6, 8	6	35.2%	No
Nausea with vomiting	moderate	10	1	5.9%	No
Nettle rash	moderate	10	1	5.9%	No
Cat allergy reactions	mild	24	1	5.9%	No
Swollen lips	mild	10	1	5.9%	No
Gastroenteritis	moderate	8	1	5.9%	No
Cat bite (hand)	severe	2	1	5.9%	Possible
Chronic urticaria (strong pruritus and wheal formation)	moderate	10	1	5.9%	Possible

## Abbreviations

Ab	Antibody
AE	Adverse Event
CSR	Clinical Study Report
eCRF	Electronic Case Report Form
ELISA	Enzyme-linked immunosorbent assay
Fel d 1	Felis domesticus 1 allergen
Fel-CuMV	Fel d 1 - Cucumber mosaic virus-like particle conjugate vaccine
GCP	Good Clinical Practice
GSS	General montly Symptom Score
GWSS	General Weekly Symptom Score
ICH	International Conference on Harmonization
IEC	Independent Ethics Committee
ITT	Intention to Treat
KEK	Cantonal Ethics Committee Zürich
LOCF	Last Observation Carried Forward
LPLV	Last Patient Last Visit
OSSS	Organ Specific Symptom Score
PP	Per Protocol
SAE	Serious Adverse Event
VAS	Visual Analogue Scale
